# Both CYP2C19 and PON1 Q192R Genotypes Influence Platelet Response to Clopidogrel by Thrombelastography in Patients with Acute Coronary Syndrome

**DOI:** 10.1155/2019/3470145

**Published:** 2019-07-18

**Authors:** Wenxing Peng, Xiujin Shi, Xiaoyu Xu, Yang Lin

**Affiliations:** ^1^Department of Pharmacy, Beijing Anzhen Hospital, Capital Medical University, Beijing, China; ^2^Beijing Institute of Heart, Lung and Blood Vessel Diseases, Beijing, China

## Abstract

**Objective:**

The objective of this study is to explore the relationships of the effects of CYP2C19 and PON1 Q192R polymorphism on the activity of clopidogrel and the risk of high platelet responsiveness (HPR) by thrombelastography in patients with acute coronary syndrome (ACS).

**Methods:**

459 ACS patients with aspirin and clopidogrel were enrolled in this observational case control study from July 13, 2015, to November 11, 2017. The patients with <30% platelet inhibition were defined as HPR group, while the others were defined as normal platelet responsiveness (NPR) group. The genotypes distribution between the groups was assessed, and the clinical impact of genetic variants was investigated by comparing the relationship between the risk of HPR and genotypes including CYP2C19⁎2, CYP2C19⁎3, CYP2C19⁎17, ABCB1, and PON1.

**Results:**

Compared with CYP2C19⁎1/⁎1 wild type carriers, CYP2C19⁎2 and ⁎3 carriers showed a significant association with the lower platelet inhibition (P=0.048). The platelet inhibition in carriers of at least one CYP2C19 loss-of-function (LOF) alleles was obviously higher than noncarriers (P=0.031). The platelet inhibition of PON1 192R carriers was lower than PON1 192Q carriers (P=0.044). Patients with the CYP2C19⁎2 and ⁎3 alleles had a greater risk of HPR than CYP2C19 wild type carriers (adjusted P=0.018 and adjusted P=0.005). At least one PON1 192R carrier predicted a significantly higher risk of HPR than PON1 192Q carriers (adjusted P=0.021). Individual CYP2C19⁎17 and ABCB1 variants did not differ significantly between the two groups.

**Conclusions:**

CYP2C19 and PON1 Q192R variants influence ADP-induced platelet inhibition by thrombelastography (TEG) in ACS patients with clopidogrel. In addition, both LOF CYP2C19 and PON1 192R variants are independent risk factors of HPR, which is measured by the relative platelet inhibition.

## 1. Introduction

Dual antiplatelet therapy with aspirin and clopidogrel has been routinely recommended in patients with acute coronary syndrome (ACS) to prevent atherothrombotic events [1, 2]. However, there are significant interindividual differences in antiplatelet effect and response to clopidogrel. Recent studies have showed that about 4%-30% of patients with the routine dose of clopidogrel cannot achieve the expected antiplatelet response [3]. This phenomenon is defined as high platelet responsiveness (HPR), and HPR has been linked to ischemic events such as death, reinfarction, or stent thrombosis [4].

Clopidogrel, an inactive prodrug, is converted into the active metabolite by two biotransformation steps. This active metabolite can irreversibly inhibit the ADP P2Y12 receptor [5]. There are many mechanisms leading to a poor response to clopidogrel, including lack of compliance, obesity, diabetes mellitus, and gene polymorphism. There is growing evidence that the cytochrome P450 2C19 (CYP2C19) is a key enzyme in the metabolization. And genes associated with CYP2C19 enzyme are expressed polymorphically. Some of CYP2C19 variants, including CYP2C19*∗*2 and *∗*3, are considered loss-of-function (LOF) alleles of CYP2C19 because they decrease the activity of enzyme. And the LOF alleles have been associated with higher platelet aggregation induced by adenosine diphosphate (ADP) [6–9]. The gain-of-function allele CYP2C19*∗*17 has been associated with ultrarapid metabolic enzyme activity. Moreover, ACS patients carrying LOF variant alleles have an increased risk of atherothrombotic events [10, 11].

In addition, there are other genes that may also affect the antiplatelet activity of clopidogrel. Some of them participate in absorption and bioactivation of clopidogrel. Paraoxonase-1 (PON1) has been considered to be a significant enzyme in the second step metabolism of clopidogrel. PON1 Q192R polymorphism is related with the wide variation of paraoxonase activity. Patients carrying PON1 Q showed lower PON1 activity and a higher risk of stent thrombosis than PON1 R [12, 13]. Unlike CYP2C19*∗*2 or *∗*3, the effect of PON1 on the response to clopidogrel was still controversial due to negative results of PON1 Q192R from some studies [14–18]. ATP-binding cassette B1 (ABCB1) may be involved in drug resistance by decreasing absorption of clopidogrel through intestinal endothelial membrane.

By now, the allele frequency of CYP2C19*∗*2 was much higher in the Asian population (35%) than Africans and Caucasians (15%) [19]. Therefore, the prevalence of HPR due to LOF CYP2C19 alleles might be particularly higher in the Asian population. The polymorphisms of CYP2C19 played a partial role in response variability to clopidogrel therapy even though it is common in the Asian population. The genetic polymorphisms of other genes, including PON1 (Q192R) and ABCB1 (C3435T), remain controversial in the Asian population [20].

The aim of our study is to further evaluate the contribution of CYP2C19 polymorphisms and still controversial gene polymorphisms of PON1 and ABCB1 in Chinese ACS patients undergoing percutaneous coronary intervention. And we applied a new platelet function test thrombelastography (TEG) to explore the relevance between gene polymorphisms and platelet responsiveness.

## 2. Methods

### 2.1. Study Population

ACS patients admitted to the Anzhen Hospital of Capital Medical University from July 13, 2015, to November 11, 2017, were enrolled in our observational case control study. The study had been approved by the Medical Clinical Research Ethics Committee of Being Anzhen Hospital. According to the American Heart Association/American College of Cardiology (AHA/ACC) criteria, the diagnosis of ACS included unstable angina (UA), non-ST segment elevation myocardial infarction (NSTEMI), and ST-elevation myocardial infarction (STEMI). Consecutive patients were assessed for eligibility for enrollment based on the following inclusion criteria: are more than 18 years old and had received 100mg aspirin daily and 75mg clopidogrel daily for at least 3 days. The exclusion criteria were as follows: contraindication to clopidogrel or aspirin, active bleeding or bleeding diseases, hematologic disorder, severe hepatic or renal insufficiency (Ccr < 30 mL/min), total platelet count less than 100×10^9^/L, and concomitant administration of other antiplatelet or anticoagulation agents.

### 2.2. Thromboelastograph with Platelet Mapping

Blood was collected at least 3 days after the administration of the first clopidogrel 75mg. These samples were sent to the laboratory and TEG test was performed within 2 hours by Hemostasis Analyzer (Haemoscope, USA). Modified TEG test utilized four channels to detect effects of antiplatelet activity via arachidonic acid (AA) and adenosine diphosphate (ADP) activators. The platelet inhibition rate of clopidogrel was calculated: [(MA_THROMBIN_-MA_ADP_)/(MA_THROMBIN_-MA_FIBRIN_)]×100%, where MA_ADP_ is the ADP-induced clot strength (which inhibits the ADP pathway of platelet activation), MA_FIBRIN_ is the fibrin-induced clot without platelet activation (measurement of fibrin contribution), and MA_THROMBIN_ is the thrombin-induced clot strength (maximum platelet activation). Low response to clopidogrel was defined as ADP-induced platelet inhibition rate of less than 30% [21].

### 2.3. Genotype Testing

Genome DNA was extracted from leucocytes of peripheral blood and stored in 3ml ethylenediaminetetraacetic acid-anticoagulated vacuum tubes. The following 5 single-nucleotide polymorphisms (SNPs) were selected: CYP2C19*∗*2 (rs4244285), CYP2C19*∗*3 (rs4986893), CYP2C19*∗*17 (rs12248560), ABCB1 (rs1045642), and PON1 (rs662). Fluorescence in situ hybridization was used for SNP genotyping with the fluorescence detector (TL988A, Xi'an TianLong). The whole process was conducted according to the manufacturer's instructions.

### 2.4. Statistical Analysis

All statistical analyses were performed with SPSS 17.0. The Kolmogorov-Smirov testing method was used to assess whether continuous data were normal distribution or not. And continuous data of normal distribution were presented as means±SD, and categorical data were presented as counts and percentages. The frequency of genotypes was classified as the presence of 0, 1, or 2 variant alleles. All SNPs in our study were tested for deviation from Hardy-Weinberg equilibrium with chi-square test. Each of genotypes was analyzed by the ANOVA test, and the intergroup difference was compared by chi-square test. Confounding factors were adjusted by logistic regression analysis. Results were expressed as odds ratio (OR), along with 95% confidence intervals (CI). A two-sided p<0.05 was considered as statistical significance.

## 3. Results

### 3.1. Characteristics of the Patients

A total of 459 eligible Chinese patients with ACS were enrolled (303 UA, 66 NSTEMI, and 90 STEMI). All patients were divided into two groups according to ADP-induced platelet inhibition rate. And ADP-induced platelet inhibition rate of less than 30% is defined as HPR group, while others are normal platelet responsiveness (NPR) group. 275 patients were categorized as HPR group, and 184 were categorized as NPR group. The mean age of all patients was 59.6. The basic data of two groups are showed in Table 1. There were no significant differences among age, body mass index (BMI), incidence of hypertension, diabetes mellitus (DM), dyslipidemia, previous PCI, current smoking, PCI due to ACS, and concomitant medications between the two groups. Meanwhile, the male had the lower risk of poor response to clopidogrel than female (P<0.001).

### 3.2. Genotypes Distribution

Genotype distribution and allele frequencies of the genetic variations were showed in Table 2. All genotype distribution was coincident with Hardy-Weinberg equilibrium. Among the 459 patients, 50.3% of them were CYP2C19*∗*2 carriers (39.2% heterozygote and 11.1% homozygote), 9.4% were CYP2C19*∗*3 carriers (no homozygote), 2.6% were CYP2C19*∗*17 carriers (2.4% heterozygote and 0.2% homozygote), 86.2% were carriers of PON1 Q192R (48.1% heterozygote and 38.1% homozygote) and 63.4% had at least one ABCB1 T (49.5% heterozygote and 13.9% homozygote).

### 3.3. Genetic Influence on Platelet Inhibition Rate

**Figure 1:**
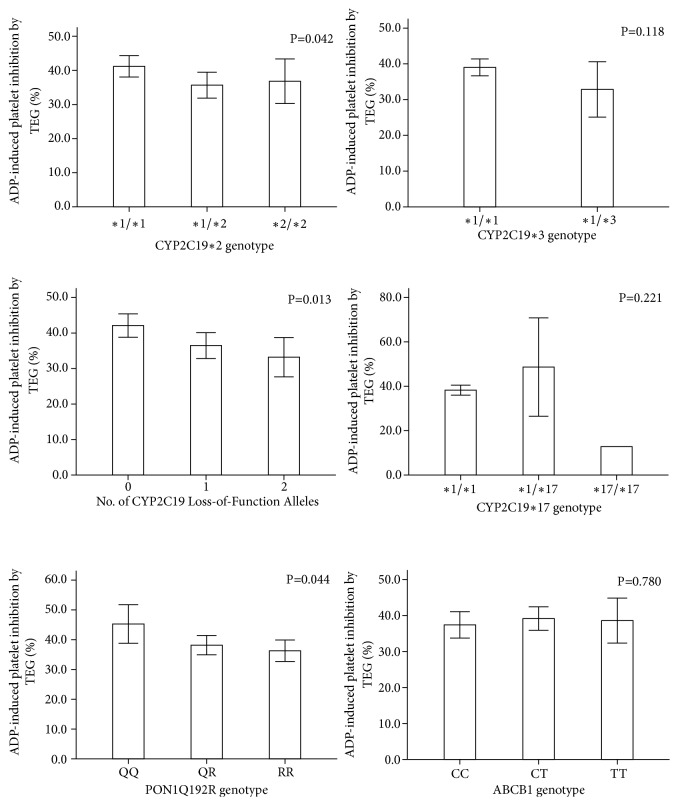
Association of clopidogrel genotypes and ADP-induced platelet inhibition by TEG. The top of each bar indicates the average of ADP-induced platelet inhibition. The whiskers above and below the bar indicate 95% CI.

Figure 1 showed the correlation between several genotypes and ADP-induced platelet inhibition by TEG. Patients with CYP2C19 *∗*1/*∗*2 had lower platelet inhibition than CYP2C19 *∗*1/*∗*1(35.66±25.74 vs. 41.33±23.92, P=0.042). There was no statistically significant difference between platelet inhibition of CYP 2C19 *∗*2/*∗*2, 1/*∗*3 and CYP2C19 *∗*1/*∗*1, but CYP 2C19 *∗*2/*∗*2, 1/*∗*3 showed a trend of lower platelet inhibition. Moreover, carriers of at least one LOF CYP2C19 allele (*∗*1/*∗*2, *∗*1/*∗*3, *∗*2/*∗*2, and *∗*2/*∗*3) showed obviously lower platelet inhibition than noncarriers (35.65±25.06 vs. 42.10 ±23.50, P=0.005). ADP-induced platelet inhibition showed lower tendency in 229 patients with PON1 192R than PON1 192Q (P=0.044), and PON1 192R homozygote (RR) had significant lower platelet inhibition than PON1 192Q homozygote (QQ) (36.30±24.16 vs. 45.28±25.69, P=0.014). Results of platelet inhibition were similar in various CYP2C19*∗*17 and ABCB1 genotypes.

### 3.4. Correlation between Genotypes and Platelet Inhibition

There was substantial individual variability in ADP-induced platelet inhibition by TEG. According to previous reported studies, the defined cutoff value for HPR was as 30% platelet inhibition. The patients who were <30% platelet inhibition were defined as HPR group, while the others were defined as NPR group. There were 275 patients (53.4%) in HPR group. Logistic regression model was applied to adjust confounding factor of gender. The relationship between genotypes and platelet inhibition was showed in Table 3. CYP2C19*∗*1/*∗*2 and *∗*1/*∗*3 had a higher risk of HPR than CYP2C19 *∗*1/*∗*1 (adjusted OR=1.607; 95%CI 1.087-2.378, P=0.018, and adjusted OR=1.483; 95%CI 1.125-1.956, P=0.005). The carriers with at least one LOF CYP2C19 alleles also showed a significant greater risk of HPR than noncarriers (adjusted OR=1.846; 95%CI 1.243-2.741, P=0.002). The patients with at least one PON1 192R had a higher risk of HPR than PON1 QQ (adjusted OR=2.033; 95%CI 1.112-3.718 P=0.021) although the PON1 heterozygote QR did not show significant results. However, neither CYP2C19*∗*17 or ABCB1 C3435T had significant independent effect in HPR.

## 4. Discussion

The recent studies provided reliable evidence that LOF CYP2C19 alleles were related with a higher risk of HPR and increased adverse cardiovascular events in ACS patients with aspirin and clopidogrel [22, 23]. The prevalence of the genotype variations in the present study was a representative of Chinese population. The frequency of CYP2C19*∗*2 was significantly higher in the Asian population than other populations. CYP2C19*∗*3 was significantly higher than in the Caucasian population. Furthermore, the frequency of CYP2C19*∗*17 in Chinese population was far lower than that reported in Caucasians and Ethiopians.

As we know, most studies evaluated the platelet function by monitoring platelet aggregation, a traditional measure of platelet function. We applied a new platelet function monitoring method to explore the influence of clopidogrel genotypes in platelet responsiveness. TEG platelet mapping assay enables achieving quantitative analysis of platelet function and has low analytical variation and better reproducibility than traditional platelet aggregation tests such as LTA and VerifyNow. In fact, there is a better correlation between the paired parameters evaluated by VerifyNow P2Y12 and the gold standard LTA, at the levels of both clopidogrel and aspirin responses, than TEG [24, 25]. But TEG is a reliable test of clotting responses to antiplatelet therapy with minimal intra- and interindividual variability. It can provide a graphic record of the physical shape of a clot during fibrin formation and subsequent lysis which was widely used in China. Moreover, TEG can provide a good correlation with ischemic cardiovascular events [26, 27]. Thus, we considered TEG could provide reliable results of the platelet function. A previous research demonstrated that ADP-induced platelet inhibition <30% could predict the combined ischemic outcome in patients with clopidogrel [21]. Thus, in this study, we defined 30% as the cutoff of HPR.

CYP2C19 is an important enzyme of the CYP family that converts clopidogrel into an active metabolite. LOF CYP2C19 alleles decrease enzymatic activities. The CYP2C19*∗*2 variant, caused by single base exchange exon 5, results in a splice site defect, therefore leading to clopidogrel resistance [28]. The CYP2C19*∗*3 variant, located within exon 4, leads to the formation of a premature stop codon and the synthesis of aberrant nonfunctional CYP2C19 protein [29]. CYP2C19*∗*2 and *∗*3 are the most common LOF variants. The CYP2C19*∗*17 variant is located in the promoter region and there is evidence that it is associated with increased gene expression and enhanced clopidogrel effect [30]. And CYP2C19*∗*17 is the most frequent gain-of-function allele in Chinese ACS patients. Thus, our study covered the three variant alleles. CYP2C19*∗*2 allele was significantly associated with lower platelet inhibition, which is in accordance with previous studies. But we failed to show a significant association in CYP2C19*∗*3 allele because of the small sample size (n=43). However, we found gender is a relevant factor of HPR and we adjusted gender by cox logistic regression test. We demonstrated an obviously higher risk of HPR in CYP2C19*∗*3 allele and at least one LOF CYP2C19 allele than CYP2C19*∗*1/*∗*1 after adjustment of gender. This result warned us that combined effect of CYP2C19*∗*2 and *∗*3 should be considered when using clopidogrel; otherwise we might ignore the influence of CYP2C19*∗*3. In the addition, we did not demonstrate a significant association between the CYP2C19*∗*17 and platelet inhibition or the risk of HPR because of a few populations of CYP2C19*∗*17 (n=12).

Some recent studies suggested that PON1 might be a significant enzyme that catalyzed the biotransformation of clopidogrel into its active metabolite, and PON1 Q192R genotypes were associated with platelet response to clopidogrel [14, 31]. However, several subsequent studies failed to replicate the influence of PON1 Q192R genotypes on platelet responsiveness, in which platelet function was evaluated by platelet aggregation tests [15, 16]. The influence of PON1 remains controversial. It has been reported that HPR defined by platelet aggregation tests was related with atherothrombotic disease [21, 22]. But relative platelet inhibition provides a novel and repeatable measure of monitoring clopidogrel responsiveness. Our study indicated PON1 192R is a risk factor for clopidogrel resistance, which is different from the previous studies. As we know, PON1 Q192R polymorphism accounts for the paraoxonase activity in serum, and PON1 192Q allele exhibits lower paraoxonase activity. However, it has been proposed that although carrying PON1 192Q allele exhibits low paraoxonase activity, its activity may be enough to efficiently metabolize clopidogrel to its final active metabolite [32]. Although PON1 is involved in the second step metabolism of clopidogrel, the metabolite of PON1 is a minor isomer instead of the active metabolite product of clopidogrel. Thus, PON1 192Q may not be an important factor in platelet function and clopidogrel. In addition, the frequency of the PON1 192Q allele in Chinese patients is relatively lower than in Caucasians. Some previous studies showed that PON1 192Q carriers had a higher risk of major adverse cardiac events (MACE) than PON1 192R. A study by Ferretti G et al. showed that an increase in activity of paraoxonase was related with a lipid-independent clinical benefit to prevent cardiovascular outcomes [33]. Therefore, we considered that this influence of the PON1 genotype may be a clopidogrel-unrelated influence including potential antioxidant and atheroprotective effects of Q192R, which has been demonstrated in some vitro studies [34].

A large number of clinical studies have demonstrated the critical role of the CYP2C19*∗*2 allele on clopidogrel responsiveness and increased risk for cardiovascular ischemic events in patients with stenting [10, 11]. ARMYDA-PRO study showed that high pre-PCI platelet reactivity might predict 30-day events, and pre-PCI platelet reactivity levels in the fourth quartile were associated with 6-fold increased risk of 30-day MACE (OR=6.1; 95%CI 1.1-18.3, p=0.033). Tang XF et al. demonstrated that HPR measured by LTA and TEG was significantly associated with MACE in Chinese patients [35].

As other observational studies, this study also had inherent limitations. First, the observational study may have the risk of selection bias. And ischemic cardiovascular events were not the major outcome in our research, with an alternative outcome TEG. Moreover, this study was single center; a larger sample and long-term follow-up should be considered in our future study. Furthermore, there is no HPR risk assessment in CYP2C19*∗*3/*∗*3 and CYP2C19*∗*17/*∗*17 because of the small sample size.

## 5. What Is New and Conclusion

In conclusion, CYP2C19 and PON1 Q192R genotypes influence ADP-induced platelet inhibition by TEG in ACS patients with clopidogrel. In addition, LOF CYP2C19 alleles and PON1 192R genotypes are independent risk factors of HPR, which is measured by the relative platelet inhibition.

## Figures and Tables

**Table 1:** Baseline characteristics of the study population.

Variables	Total(n=459)	NPR (n=275)	HPR (n=184)	*P*
*Age (years)*	59.58±10.28	59.35±10.47	59.93±10.00	0.551
*Gender (male)n(*%)	350(76.3%)	229(83.3%)	121(65.8%)	<0.001
*BMI (kg/m* ^*2*^)	26.15±3.64	25.94±3.38	26.32±4.01	0.318
*Risk factor n(*%)				
Hypertension	264(57.5%)	150(54.5%)	114(62.0%)	0.115
DM	149(32.5%)	94(34.2%)	55(29.9%)	0.336
Dyslipidemia	189(41.2%)	117(42.5%)	72(39.1%)	0.466
*Previous PCI*	85(18.5%)	52(18.9%)	33(17.9%)	0.792
*Current smoking*	168(36.6%)	110(40.0%)	58(31.5%)	0.065
*Concomitant medications(*%)				
Statin	454(98.9%)	272(98.9%)	182(98.9%)	0.997
ACEI/ARB	305(66.4%)	190(70.2%)	112(60.9%)	0.069
*β*-blocker	381(83.0%)	224(81.5%)	157(85.3%)	0.279
PPI	294(64.1%)	185(67.5%)	109(58.9%)	0.060
*PCI due to ACS(*%)	331(72.1%)	196(71.3%)	135(73.4%)	0.623
*Laboratory measurement*				
Creatinine clearance(mL/min)∗	88.51(71.30,109.28)	88.35(70.32,108.19)	89.35(72.30,110.74)	0.670
Glucose(mg/dl)∗	5.54(5.02,6.84)	5.51(4.99,6.70)	5.57(5.07,7.17)	0.386
Triglyceride(mmol/L)∗	1.48(1.05,2.11)	1.48(1.03,2.05)	1.48(1.07,2.21)	0.408
Cholesterol(mmol/L)	4.11±1.01	4.13±0.95	4.09±1.11	0.105
HDL(mmol/L)∗	0.95(0.80,1.09)	0.95(0.80,1.08)	0.94(0.81,1.11)	0.703
LDL(mmol/L)	2.49±0.87	2.52±0.81	2.45±0.96	0.055
PLT(×10^9^/L)	212.9±56.0	212.0±54.6	214.5±58.2	0.063

*∗*These risk factors were not in accord with normal distribution.

**Table 2:** Genotype distribution among the study population.

		Total (n=459)	Minor Allele Frequency(%)	Hardy-Weinberg equilibrium (P value)
CYP2C19*∗*2	GG(*∗*1/*∗*1)	228(49.7%)	30.7	0.941
	GA(*∗*1/*∗*2)	180(39.2%)		
	AA(*∗*2/*∗*2)	51(11.1%)		
CYP2C19*∗*3	GG(*∗*1/*∗*1)	416(90.6%)	4.7	0.592
	GA(*∗*1/*∗*3)	43(9.4%)		
	AA(*∗*3/*∗*3)	0(0%)		
CYP2C19*∗*17	CC(*∗*1/*∗*1)	447(97.4%)	1.4	0.558
	CT(*∗*1/*∗*17)	11(2.4%)		
	TT(*∗*17/*∗*17)	1(0.2%)		
PON1	QQ(AA)	63(13.7%)	37.8	0.927
	QR(AG)	221(48.1%)		
	RR(GG)	175(38.1%)		
ABCB1	CC	168(36.6%)	38.7	0.802
	CT	227(49.5%)		
	TT	64(13.9%)		

**Table 3:** Relationship between genotypes and platelet inhibition.

Variant genotype SNP		NPR (n=275)	HPR (n=184)	Odds Ratio(95%CI)	P value	Adjusted Odds Ratio*∗*(95%CI)	P value*∗*
CYP2C19*∗*2	GA(*∗*1/*∗*2)	97(35.3%)	83(45.1%)	1.508(1.030,2.208)	0.034	1.607(1.087,2.378)	0.018
	AA(*∗*2/*∗*2)	29(10.5%)	22(12.0%)	1.152(0.639,2.075)	0.637	1.090(0.807,1.471)	0.575
	At least 1	126(45.8%)	105(57.1%)	1.572(1.079,2.289)	0.018	1.696(1.151,2.498)	0.008
CYP2C19*∗*3	GA(*∗*1/*∗*3)	21(7.6%)	22(12.0%)	1.643(0.875,3.083)	0.120	1.483(1.125,1.956)	0.005
	AA(*∗*3/*∗*3)	0	0	-	-	-	-
	At least 1	21(7.6%)	22(12.0%)	1.643(0.875,3.083)	0.120	1.483(1.125,1.956)	0.005
CYP2C19 LOF allele number	1	108(39.3%)	89(48.4%)	1.449(0.994,2.112)	0.054	1.525(1.036,2.245)	0.032
	2	34(12.4%)	30(16.3%)	1.381(0.812,2.348)	0.232	1.430(0.832,2.459)	0.195
	At least 1	142(51.6%)	119(64.8%)	1.715(1.168,2.517)	0.006	1.846(1.243,2.741)	0.002
CYP2C19*∗*17	CT(*∗*1/*∗*17)	7(2.5%)	4(2.2%)	0.851(0.245,2.949)	0.799	0.896(0.253,3.174)	0.865
	TT(*∗*17/*∗*17)	0	1(0.5%)	-	-	-	-
	At least 1	7(2.5%)	5(2.7%)	1.069(0.334,3.422)	0.910	1.153(0.353,3.763)	0.813
PON1	QR(AG)	126(45.8%)	95(51.6%)	1.262(0.868,1.835)	0.222	1.304(0.889,1.911)	0.174
	RR(GG)	103(37.5%)	72(39.1%)	1.074(0.731,1.576)	0.717	1.053(0.711,1.558)	0.798
	At least 1R	229(83.3%)	167(90.8%)	1.973(1.093,3.563)	0.022	2.033(1.112,3.718)	0.021
ABCB1	CT	137(49.8%)	90(48.9%)	0.964(0.664,1.401)	0.849	0.971(0.663,1.421)	0.878
	TT	36(13.1%)	28(15.2%)	1.192(0.699,2.031)	0.519	1.273(0.738,2.193)	0.386
	At least 1T	173(62.9%)	118(64.1%)	1.054(0.715,1.554)	0.790	1.099(0.739,1.634)	0.642

*∗* Adjusted risk factor of gender by logistic regression model.

## Data Availability

The data used to support the findings of this study are available from the corresponding author upon request.

## References

[1] Ibanez B., James S., Agewall S. (2018). 2017 ESC Guidelines for the management of acute myocardial infarction in patients presenting with ST-segment elevation: The Task Force for the management of acute myocardial infarction in patients presenting with ST-segment elevation of the European Society of Cardiology (ESC). *European Heart Journal*.

[2] Valgimigli M., Bueno H., Byrne R. A. (2018). 2017 ESC focused update on dual antiplatelet therapy in coronary artery disease developed in collaboration with EACTS: The task force for dual antiplatelet therapy in coronary artery disease of the European Society of Cardiology (ESC) and of the European Association for Cardio-Thoracic Surgery (EACTS). *European Heart Journal*.

[3] Sugunaraj J. P., Palaniswamy C., Selvaraj D. R. (2010). Clopidogrel resistance. *American Journal of Therapeutics*.

[4] Patti G., Nusca A., Mangiacapra F. (2008). Point-of-care measurement of clopidogrel responsiveness predicts clinical outcome in patients undergoing percutaneous coronary intervention results of the armyda-pro (antiplatelet therapy for reduction of myocardial damage during angioplasty-platelet reactivity predicts outcome) study. *Journal of the American College of Cardiology*.

[5] Turner R. M., Pirmohamed M. (2014). Cardiovascular pharmacogenomics: expectations and practical benefits. *Clinical Pharmacology & Therapeutics*.

[6] Arima Y., Hokimoto S., Akasaka T. (2015). Comparison of the effect of CYP2C19 polymorphism on clinical outcome between acute coronary syndrome and stable angina. *Journal of Cardiology*.

[7] Hou X., Shi J., Sun H. (2014). Gene polymorphism of cytochrome P450 2C19∗2 and clopidogrel resistance reflected by platelet function assays: a meta-analysis. *European Journal of Clinical Pharmacology*.

[8] Marchini J. F., Pinto M. R., Novaes G. C. (2017). Decreased platelet responsiveness to clopidogrel correlates with CYP2C19 and PON1 polymorphisms in atherosclerotic patients. *Brazilian Journal of Medical and Biological Research*.

[9] Mărginean A., Bănescu C., Moldovan V. (2017). The impact of CYP2C19 loss-of-function polymorphisms, clinical, and demographic variables on platelet response to clopidogrel evaluated using impedance aggregometry. *Clinical and Applied Thrombosis/Hemostasis*.

[10] Paré G., Mehta S. R., Yusuf S. (2010). Effects of CYP2C19 genotype on outcomes of clopidogrel treatment. *The New England Journal of Medicine*.

[11] Holmes M. V., Perel P., Shah T., Hingorani A. D., Casas J. P. (2011). CYP2C19 genotype, clopidogrel metabolism, platelet function, and cardiovascular events: a systematic review and meta-analysis. *Journal of the American Medical Association*.

[12] Chen Y., Huang X., Tang Y., Xie Y., Zhang Y. (2015). Both PON1 Q192R and CYP2C19∗2 influence platelet response to clopidogrel and ischemic events in Chinese patients undergoing percutaneous coronary intervention. *International Journal of Clinical and Experimental Medicine*.

[13] Park K. W., Park J. J., Kang J. (2013). Paraoxonase 1 gene polymorphism does not affect clopidogrel response variability but is associated with clinical outcome after PCI. *PLoS ONE*.

[14] Tresukosol D., Suktitipat B., Hunnangkul S. (2014). Effects of cytochrome P450 2C19 and paraoxonase 1 polymorphisms on antiplatelet response to clopidogrel therapy in patients with coronary artery disease. *PLoS ONE*.

[15] Zhang L., Chen Y., Jin Y. (2013). Genetic determinants of high on-treatment platelet reactivity in clopidogrel treated Chinese patients. *Thrombosis Research*.

[16] Price M. J., Murray S. S., Angiolillo D. J. (2012). Influence of genetic polymorphisms on the effect of high- and standard-dose clopidogrel after percutaneous coronary intervention: the GIFT (Genotype Information and Functional Testing) study. *Journal of the American College of Cardiology*.

[17] Lei H. P., Yu X. Y., Wu H. (2018). Effects of PON1 gene promoter dna methylation and genetic variations on the clinical outcomes of dual antiplatelet therapy for patients undergoing percutaneous coronary intervention. *Clinical Pharmacokinetics*.

[18] Ma W., Liang Y., Zhu J. (2016). Relationship of paraoxonase-1 Q192R genotypes and in-stent restenosis and re-stenting in Chinese patients after coronary stenting. *Atherosclerosis*.

[19] Scott S. A., Sangkuhl K., Stein C. M. (2013). Clinical pharmacogenetics implementation consortium guidelines for CYP2C19 genotype and clopidogrel therapy. *Clinical Pharmacology & Therapeutics*.

[20] Tang X., Wang J., Zhang J. (2013). Effect of the CYP2C19∗2 and ∗3 genotypes, ABCB1 C3435T and PON1 Q192R alleles on the pharmacodynamics and adverse clinical events of clopidogrel in Chinese people after percutaneous coronary intervention. *European Journal of Clinical Pharmacology*.

[21] Tang N., Yin S., Sun Z., Xu X., Qin J. (2015). The relationship between on-clopidogrel platelet reactivity, genotype, and post-percutaneous coronary intervention outcomes in Chinese patients. *Scandinavian Journal of Clinical & Laboratory Investigation*.

[22] Jin H.-Y., Yang T.-H., Kim D.-I. (2013). High post-clopidogrel platelet reactivity assessed by a point-of-care assay predicts long-term clinical outcomes in patients with ST-segment elevation myocardial infarction who underwent primary coronary stenting. *International Journal of Cardiology*.

[23] Rivard G. E., Brummel-Ziedins K. E., Mann K. G. (2005). Evaluation of the profile of thrombin generation during the process of whole blood clotting as assessed by thrombelastography. *Journal of Thrombosis and Haemostasis*.

[24] Lv H., Wu S., Liu X. (2016). Comparison of VerifyNow P2Y12 and thrombelastography for assessing clopidogrel response in stroke patients in China. *Neurological Sciences*.

[25] Zhang H., Kim M. H., Jeong Y. (2014). Predictive values of post-clopidogrel platelet reactivity assessed by different platelet function tests on ischemic events in East Asian patients treated with PCI. *Platelets*.

[26] Gurbel P. A., Bliden K. P., Navickas I. A. (2010). Adenosine diphosphate–induced platelet-fibrin clot strength: a new thrombelastographic indicator of long-term poststenting ischemic events. *American Heart Journal*.

[27] Bliden K. P., DiChiara J., Tantry U. S., Bassi A. K., Chaganti S. K., Gurbel P. A. (2007). Increased risk in patients with high platelet aggregation receiving chronic clopidogrel therapy undergoing percutaneous coronary intervention: is the current antiplatelet therapy adequate?. *Journal of the American College of Cardiology*.

[28] Sibbing D., Stegherr J., Latz W. (2009). Cytochrome P450 2C19 loss-of-function polymorphism and stent thrombosis following percutaneous coronary intervention. *European Heart Journal*.

[29] De Morais S. M. F., Wilkinson G. R., Blaisdell J., Nakamura K., Meyer U. A., Goldstein J. A. (1994). The major genetic defect responsible for the polymorphism of S-mephenytoin metabolism in humans. *The Journal of Biological Chemistry*.

[30] Sibbing D., Koch W., Gebhard D. (2010). Cytochrome 2C19∗17 allelic variant, platelet aggregation, bleeding events, and stent thrombosis in clopidogrel-treated patients with coronary stent placement. *Circulation*.

[31] Wu H., Qian J., Xu J. (2013). Besides CYP2C19, PON1 genetic variant influences post-clopidogrel platelet reactivity in Chinese patients. *International Journal of Cardiology*.

[32] Tselepis A. D., Tsoumani M. E., Kalantzi K. I. (2011). Influence of high-density lipoprotein and paraoxonase-1 on platelet reactivity in patients with acute coronary syndromes receiving clopidogrel therapy. *Journal of Thrombosis and Haemostasis*.

[33] Ferretti G., Bacchetti T., Sahebkar A. (2015). Effect of statin therapy on paraoxonase-1 status: a systematic review and meta-analysis of 25 clinical trials. *Progress in Lipid Research*.

[34] Paré G., Ross S., Mehta S. R. (2012). Effect of PON1 Q192R genetic polymorphism on clopidogrel efficacy and cardiovascular events in the clopidogrel in the unstable angina to prevent recurrent events trial and the atrial fibrillation clopidogrel trial with irbesartan for prevention of vascular events. *Circulation: Cardiovascular Genetics*.

[35] Tang X., Han Y., Zhang J. (2015). Comparing of light transmittance aggregometry and modified thrombelastograph in predicting clinical outcomes in chinese patients undergoing coronary stenting with clopidogrel. *Chinese Medical Journal*.

